# A parallel bioreactor strategy to rapidly determine growth-coupling relationships for bioproduction: a mevalonate case study

**DOI:** 10.1186/s13068-024-02599-x

**Published:** 2025-01-17

**Authors:** Alec Banner, Joseph Webb, Nigel Scrutton

**Affiliations:** 1 Manchester Institute of Biotechnology, The University of Manchester, 131 Princess Street, Manchester, M1 7DN UK; 2 Present Address: Imperagen Ltd, Manchester Science Park, Manchester, M15 6SE UK

**Keywords:** Mevalonate, Parallel bioreactor, Growth-coupling, Fermentation, Scale-up

## Abstract

**Background:**

The climate crisis and depleting fossil fuel reserves have led to a drive for ‘green’ alternatives to the way we manufacture chemicals, and the formation of a bioeconomy that reduces our reliance on petrochemical-based feedstocks. Advances in Synthetic biology have provided the opportunity to engineer micro-organisms to produce compounds from renewable feedstocks, which could play a role in replacing traditional, petrochemical based, manufacturing routes. However, there are few examples of bio-manufactured products achieving commercialisation. This may be partially due to a disparity between academic and industrial focus, and a greater emphasis needs to be placed on economic feasibility at an earlier stage. Terpenoids are a class of compounds with diverse use across fuel, materials and pharmaceutical industries and can be manufactured biologically from the key intermediate mevalonate.

**Results:**

Here, we report on a method of utilising parallel bioreactors to rapidly map the growth-coupling relationship between the specific product formation rate, specific substrate utilisation rate and specific growth rate. Using mevalonate as an example product, a maximum product yield coefficient of 0.18 g_p_/g_s_ was achieved at a growth rate ($\mu$) of 0.34 h^−1^. However, this process also led to the formation of the toxic byproduct acetate, which can slow growth and cause problems during downstream processing. By using gene editing to knock out the *ackA-pta* operon and *poxB* from *E. coli* BW25113, we were able to achieve the same optimum production rate, without the formation of acetate.

**Conclusions:**

We demonstrated the power of using parallel bioreactors to assess productivity and the growth-coupling relationship between growth rate and product yield coefficient of mevalonate production. Using genetic engineering, our resultant strain demonstrated rapid mevalonate formation without the unwanted byproduct acetate. Mevalonate production is quantified and reported in industrially relevant units, including key parameters like conversion efficiency that are often omitted in early-stage publications reporting only titre in g/L.

**Supplementary Information:**

The online version contains supplementary material available at 10.1186/s13068-024-02599-x.

## Background

Depleting fossil fuels reserves and climate change concerns have pushed synthetic biology to demonstrate production of a vast array of bioderived commodity and high-value compounds [1–4]. However, despite being able to bio-produce many compounds with the potential to replace fossil fuel derived counterparts, few of these production methods have reached commercialisation [5].

Common barriers to the commercialisation of bioprocesses are financial, regulatory and the availability of appropriate facilities [6]. In academia, the financial viability of a process is often considered secondary to the advancement of scientific knowledge. However, if more processes are to reach commercialisation, whether through the growth of spinout companies or investment by larger established industrial partners, the financial viability of a process needs to be taken into consideration earlier in the development cycle.

The commercial viability of a bioprocess can be studied through the use of techno economic analysis (TEA) [7], where a well-founded TEA can help to encourage and de-risk potential investment. These analyses not only look at reported titres but consider other factors such as the capital cost of building the fermentation plant (CAP-EX), ongoing operating costs (OP-EX), such as energy and raw substrate requirements, and down-stream processing [7]. All are factors that are not typically considered at the academic discovery stage.

**Fig. 1:**
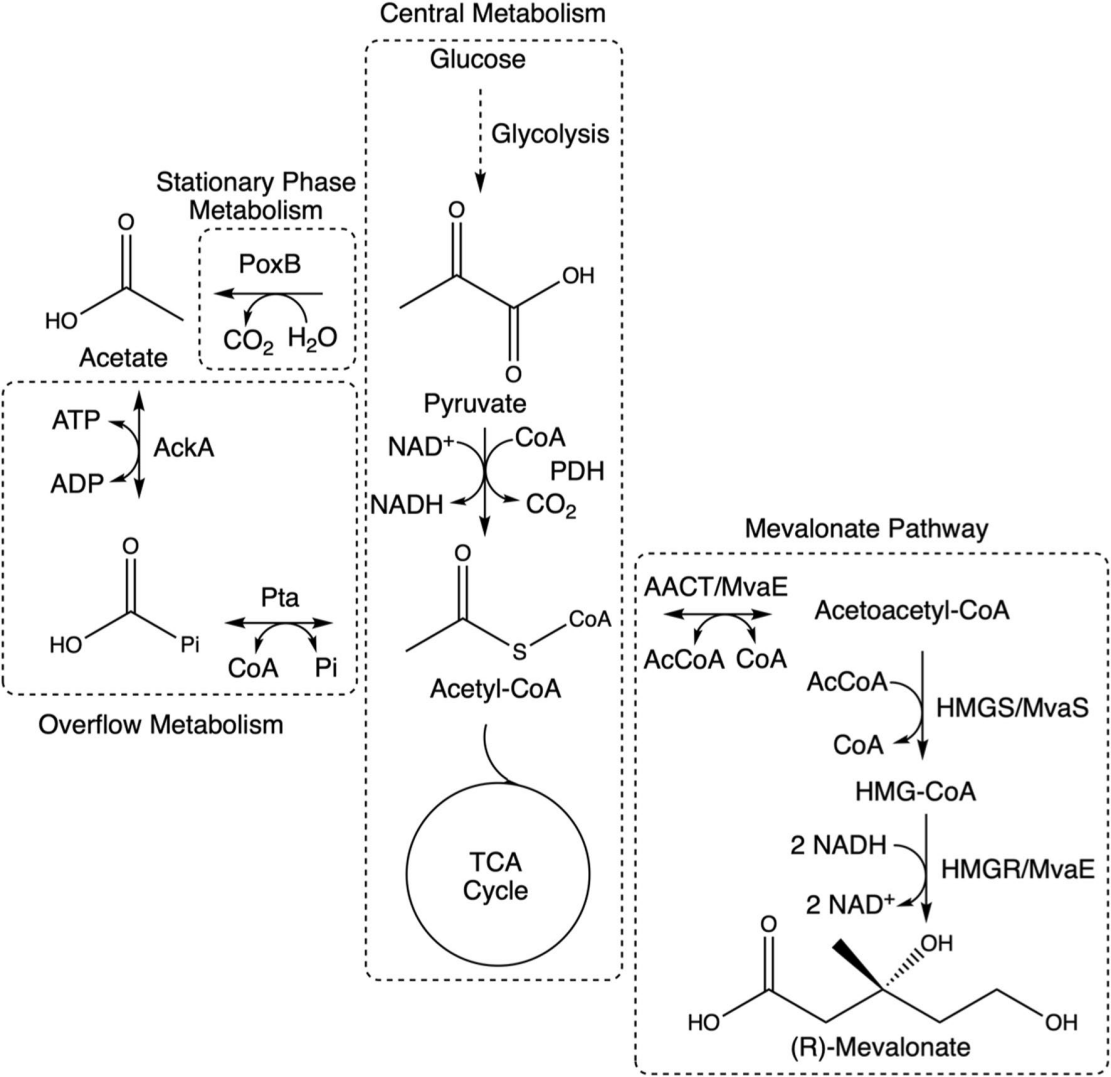
Reaction scheme of *E. coli* central metabolism, overflow metabolism, stationary phase acetate production and mevalonate production. AcCoA = acetyl-CoA, HMG-CoA = 3-hydroxy-3-methylglutaryl-CoA, MVA = mevalonate, AACT = acetoacetyl-CoA thiolase, HMGR = 3-hydroxy-3-methylglutarate reductase, HMGS = 3-hydroxy-3-methylglutarate synthase, Pta = phosphate acetyltransferase, AckA = acetate kinase, PoxB = pyruvate oxidase, TCA = tricarboxylic acid, PDH = pyruvate dehydrogenase complex

Mevalonate is a 5-carbon carboxylic acid with uses in the cosmetics industry, but mainly serves as a precursor for terpene synthesis with broad application in pharmaceutical, flavour/fragrance and fuel industries [8]. Mevalonate is produced from central metabolism by the mevalonate pathway in three enzymatic steps from acetyl-CoA (Fig. 1). Terpenes are usually found in nature as plant secondary metabolites [9]. However, when they are produced by heterologous expression of the enzymatic pathway during growth phase, they can be considered growth-coupled, primary metabolites, due to the competition for acetyl-CoA flux. The highest reported production titres of mevalonate by *E. coli* are 111.3 g/L during a 120 h, fed-batch fermentation [10] or 47 g/L in a 50 h fed-batch fermentation [11]. However, we propose that titres reported in g/L lack key information relating to time and substrate requirements, so instead study productivity via specific product formation rate (q_p_) in g_p_/g_x_/h. We also recognise the impact specific growth rate ($\mu$) has on q_p_ and product yield coefficient (Y_p/s_), so endeavour to study a full ‘growth-coupling relationship’.

Parallel bioreactors, such as the Sartorius AMBR250 system, allow multiple fermentations to be carried out simultaneously, decreasing the time required to generate data. Growth-coupling relationships are strain and condition specific, and therefore will vary for every bioprocess. It is possible to use parallel bioreactor systems to rapidly estimate the landscape of the growth-coupling relationship within a single experimental run, using calculated fed-batch feeds to achieve pseudo steady-state growth conditions, without the need for long and challenging chemostat experiments or sequential fed-batch fermentations. Parallelisation decreases the time, and therefore cost, associated with studying these relationships and could be readily implemented during academic studies. Making these data available during low technology readiness level research [12], could help navigate the landscape of technoeconomic analysis, in a cost-effective manner, when compared to titre alone which provides a single datapoint. In turn, this could help commercialisation of the vast array of compounds produced by synthetic biology.

During fermentations with excess glucose or high growth rates, *E. coli* produces acetate through so called ‘overflow’ metabolism (Fig. 1) [13]. The purpose and effects of overflow metabolism are debated, but it is thought that production of acetate may help restore cofactor availability, maintain redox balance and regenerate CoA pool from acetyl-CoA accumulation. However, acetate accumulation is toxic to the cell, affecting growth and, therefore, is undesirable during a fermentation process [14]. In addition, removal of dilute acetate from aqueous fermentation broth during downstream processing (DSP) is challenging, especially if the product of interest is an organic acid, such as mevalonate [15–17]. Therefore, we knocked out the main acetate producing pathways in *E. coli*, *poxB* and *ackA/pta* operon (Fig. 1), and showed that we can maintain the same production rates, without the build-up of a toxic and hard to separate byproduct.

## Results

### Steady-state, fed-batch mevalonate production

**Fig. 2:**
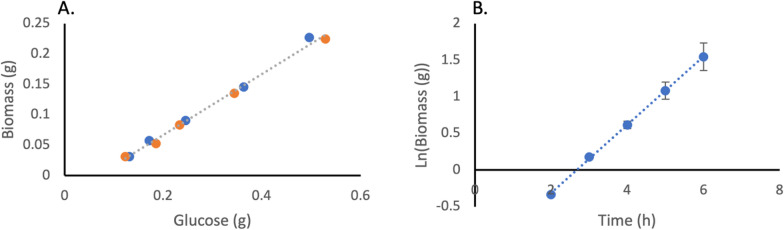
Calculation of Y_x/s_ and $\mu$_max_ of BW_MvaES (n = 2). **A**) Plot of biomass against glucose with a gradient equal to Y_X/S_ (0.5 g_x_/g_s_, root mean squared error (RMSE) = 0.0053, correlation coefficient (R^2^) = 0.994) (blue = bioreactor 1, orange = bioreactor 2). **B**) Natural logarithm of biomass against time since inoculation, where gradient gives an assumed $\mu$_max_ (0.47 h^−1^, RMSE = 0.016, R^2^ = 0.999)

An initial batch fermentation was used to calculate the biomass yield coefficient (Y_X/S_) and maximum growth rate ($\mu$_max_) of *E. coli* BW_MvaES (Fig. 2). The strain was induced to replicate the growth kinetics of the producing strain. In the batch medium, all growth nutrients are in excess, and therefore, the growth rate during the batch phase can be assumed to be $\mu$_max_.

These values were then used to calculate feeding rates (Eq. 1) to give the desired, fixed growth rates ($\mu$_set_). The feeding rates (Eq. 1) assume a constant volume, and therefore the measured rates are only true when the volume of feed added is significantly lower than the initial volume. This means that only initial rates remain constant, and as feed volume increases over time, $\mu$ tends to decrease. However, whilst the amount of feed added remains significantly smaller than the initial culture volume, the cells are generally considered to be in steady state [18]. Steady state, $\mu$_set_ values of 0.45, 0.375, 0.3, 0.225, 0.15 and 0.075 were selected to give broad coverage over biologically relevant growth rates:

$${F}_{t}=\left(\frac{{\mu }_{set}}{{Y}_{x/s}}+ms\right).\left(\frac{{x}_{0}{.v}_{0}}{{\omega }_{in}}\right). {e}^{{\mu }_{set}.t}.$$

*Equation 1 Calculating feeding rates (F*_*t*_*) to give desired *$\mu$_*set*_*. ms* = *maintenance value (0.06 g*_*s*_*/g*_*x*_*/h *[19]*), x*_*0*_ = *initial biomass concentration (g/L), v*_*0*_ = *initial culture volume (l), *$\omega_{in}$ = *concentration of rate limiting substrate in feed, t* = *time (h).*

**Fig. 3:**
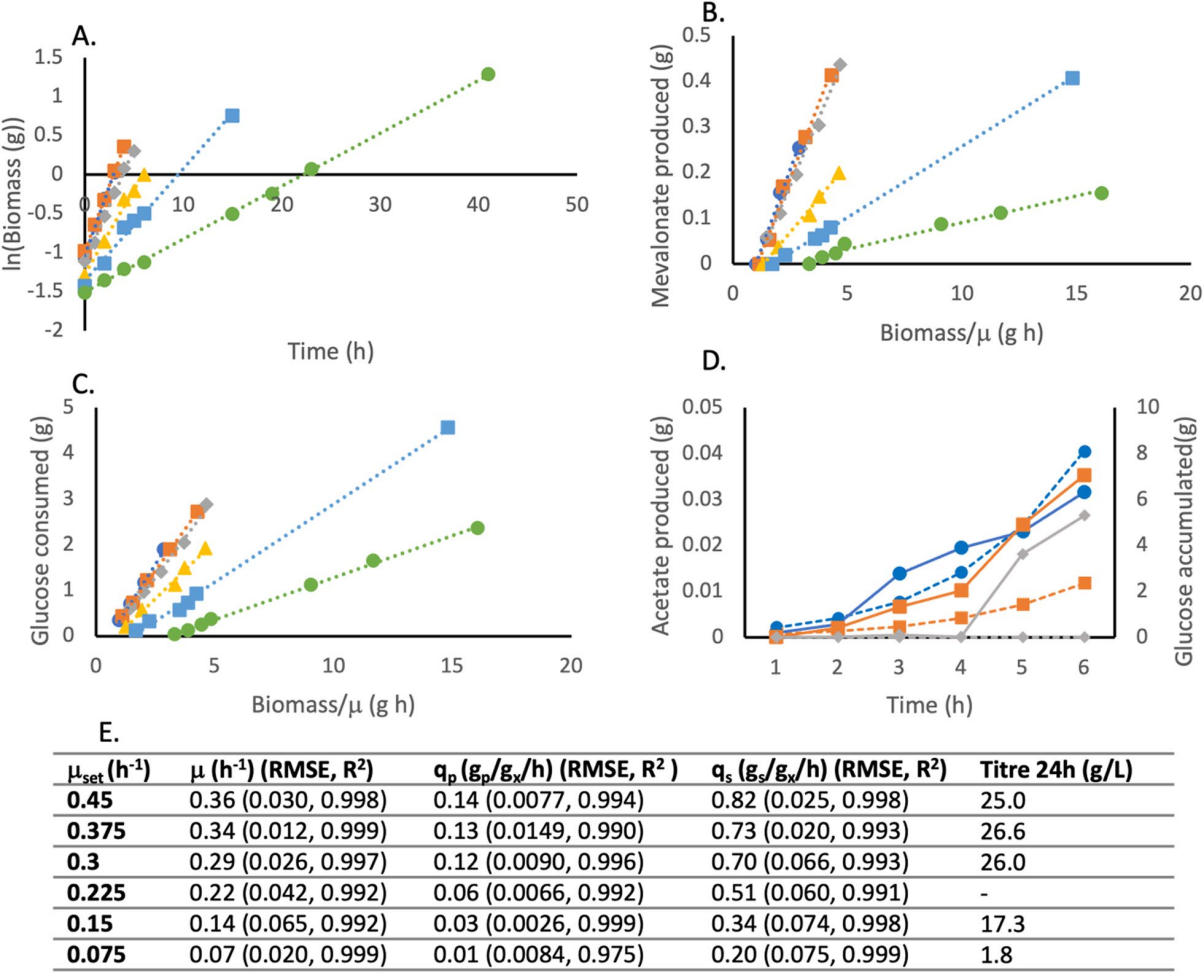
Mevalonate production by BW_MvaES, during six parallel fed-batch fermentations with defined $\mu$_set_ values. Values normalised to t = 0, for the beginning of steady-state conditions after batch phase, once feed has begun. **A** Specific growth rate of fed-batch reactions. **B** Specific product formation rate (q_p_) of fed-batch reactions. **C** Specific substrate consumption rate (q_s_) of fed-batch reactions. **D** amount of glucose and acetate present in the culture medium (based on HPLC sample analysis) (solid lines acetate, dotted lines glucose). **E** Table containing the values, RMSE and R^2^ of $\mu$, q_p_ and q_s_ for each $\mu$_set_, obtained from panels A-C, and titre after 24 h of feeding. Blue circles: $\mu$_set_ = 0.45 h^−1^, orange squares: $\mu$_set_ = 0.375 h^−1^, grey diamonds: $\mu$_set_ = 0.3 h^−1^, yellow triangles: $\mu$_set_ = 0.225 h^−1^, light blue squares: $\mu$_set_ = 0.15 h^−1^, green circles: $\mu$_set_ = 0.075 h^−1^

Initially, in all fed-batch reactions, steady-state conditions with constant growth rates were achieved (Fig. 3 A). The linearity of the plots indicates steady-state conditions and variation of q_p_ with growth rate, confirms that production is growth coupled. For the lower growth rates, the specific growth rate was equal to $\mu$_set_. However, for $\mu$_set_ 0.45 h^−1^ and 0.375 h^−1^, the specific growth rates were slightly lower than desired. This was because the glucose uptake rate was too low resulting in accumulation of glucose in the medium (Fig. 3 D). As expected, the highest specific substrate consumption rates (q_s_) were by cultures with the highest $\mu$_set_ (Fig. 3 C). The highest $\mu$_set_ also gave rise to the highest specific product formation rates (q_p_) of mevalonate, up to 0.14 g_p_/g_x_/h (Fig. 3 B). However, during growth at the highest $\mu$_set_ values (0.45, 0.375 and 0.3 h^−1^), acetate was produced and accumulated in the culture medium (Fig. 3 D).

### Growth-coupling of mevalonate production

**Fig. 4:**
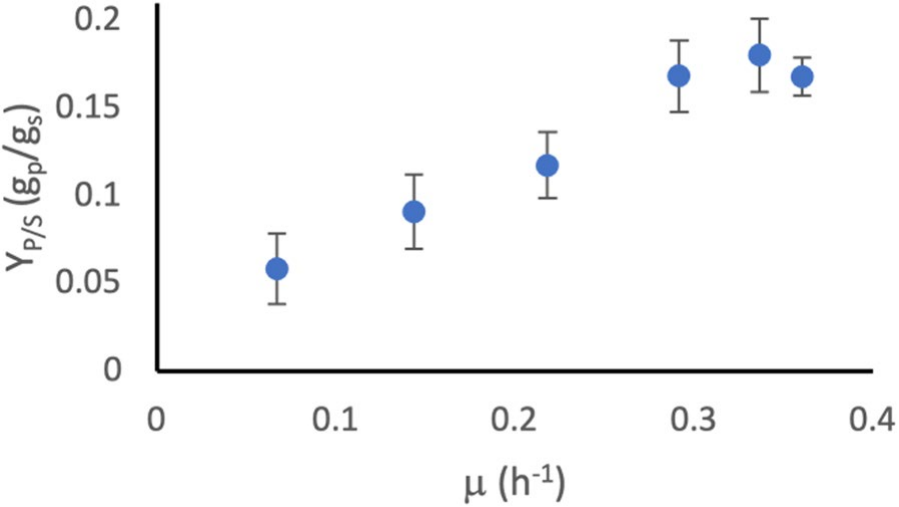
The relationship between Y_P/S_ and $\mu$for BW_MvaES. Error estimated by error propagation of q_p_ and q_s_ RMSE values

By analysing the relationship between q_s_, q_p_ and $\mu$ (obtained in Fig. 3), it was possible to determine the growth rate at which the largest proportion of substrate was diverted to product, as demonstrated by a high product yield coefficient (Y_P/s_) (Fig. 4). In the case of mevalonate production by BW_MvaES, high growth rates gave the highest Y_P/S_ (0.18 g_P_/g_S_ at 0.34 h^−1^), whereas a growth rate of 0.07 h^−1^ produced threefold less product per g substrate (0.06 g_P_/g_S_)

### Using CRISPR to engineer acetate-free, mevalonate-producing strains

**Fig. 5:**
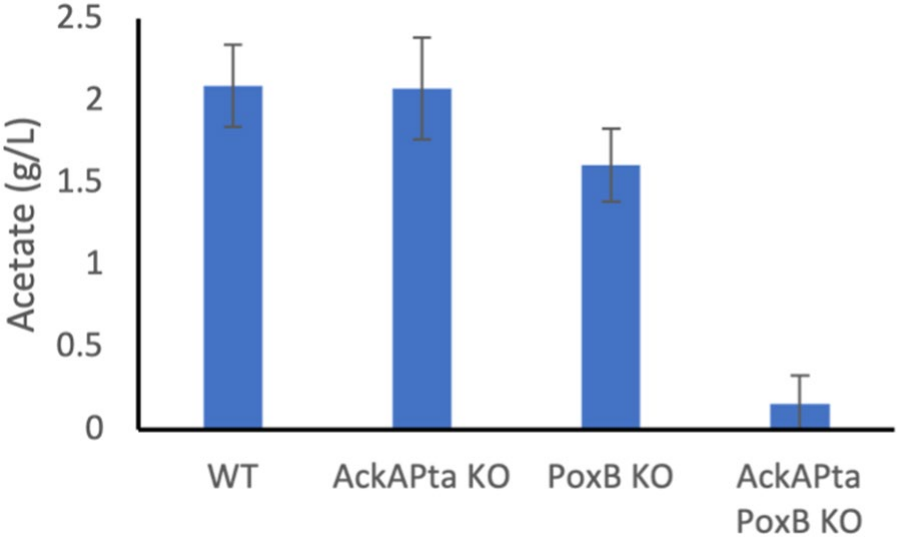
Acetate production after 24 h of batch culture in LB supplemented with 20 g/L glucose (n = 3). WT = wild type BW25113, ackA-pta KO = BW25113 ΔackA-pta, poxB KO = BW25113 ΔpoxB and ackApta poxB KO = BW25113 ΔackApta ΔpoxB

CRISPR–cas12a was used to knockout the acetate production pathways native to *E. coli* [20]. The *ackA-pta* operon is responsible for acetate production during exponential growth when glucose uptake rates are high. During stationary phase metabolism, *poxB* is responsible for most acetate produced (Fig. 1) [13]. The general effectiveness of reducing acetate formation by knocking out these pathways was assessed by growth on Luria–Bertani (LB) supplemented with 20 g/L glucose to induce acetate formation. Under these conditions, knocking out a single pathway did not remove acetate production, presumably due to compensatory metabolism allowing the cells to utilise an alternative pathway (Fig. 5). However, knocking out both *ackA-pta* and *poxB* significantly reduced acetate production by *E. coli* (Student’s t-test: p < 0.05).

### Acetate-free fed-batch, mevalonate production

**Fig. 6:**
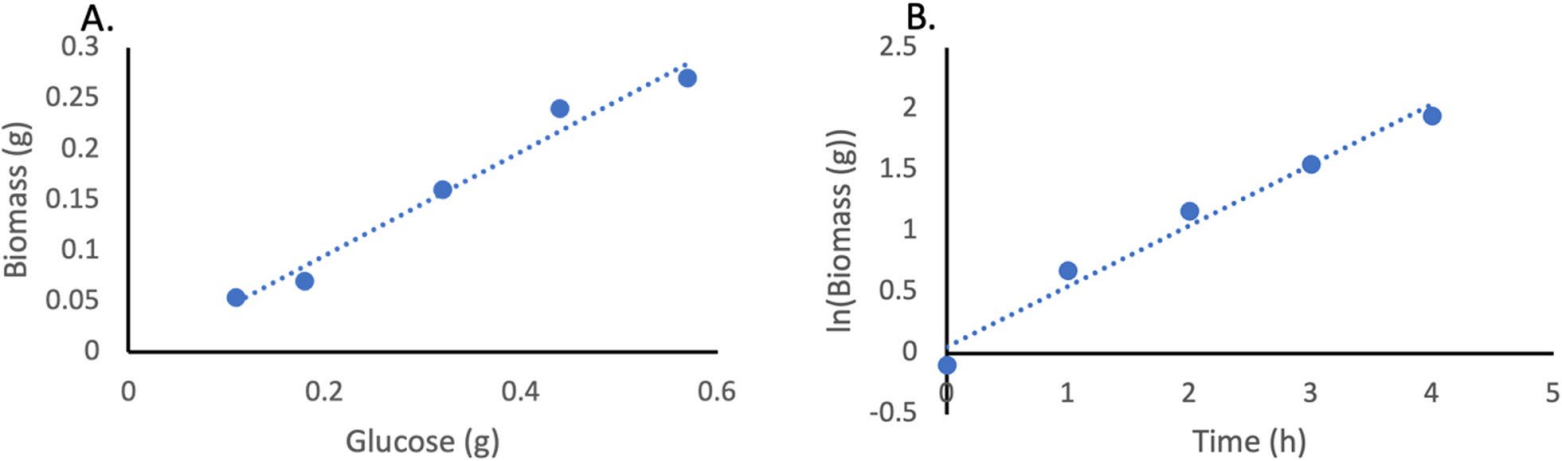
Calculation of Y_x/s_ and $\mu$_max_ of KO_MvaES. **A** Plot of produced biomass against consumed glucose with a gradient equal to Y_x/s_ (0.51 g_x_/g_s_, RMSE = 0.014, R^2^ = 0.975). **B** Natural logarithm of biomass against time, where gradient gives an assumed $\mu$_max_ (0.49 h^−1^, RMSE = 0.16, R^2^ = 0.975)

The Δ*ackApta* Δ*poxB* double knockout (KO) KO_MvaES was assessed for its ability to produce mevalonate. Batch cultures of the KO strain were used to calculate Y_X/S_ and $\mu$_max_ (Fig. 6). These were then used to determine F_t_ (Eq. 1). $\mu$_set_ values of 0.4, 0.35, 0.3, 0.25, 0.2 and 0.15 h^−1^ were selected so that g_s_/g_x_/h could be measured across a range of relevant growth rates.

**Fig. 7:**
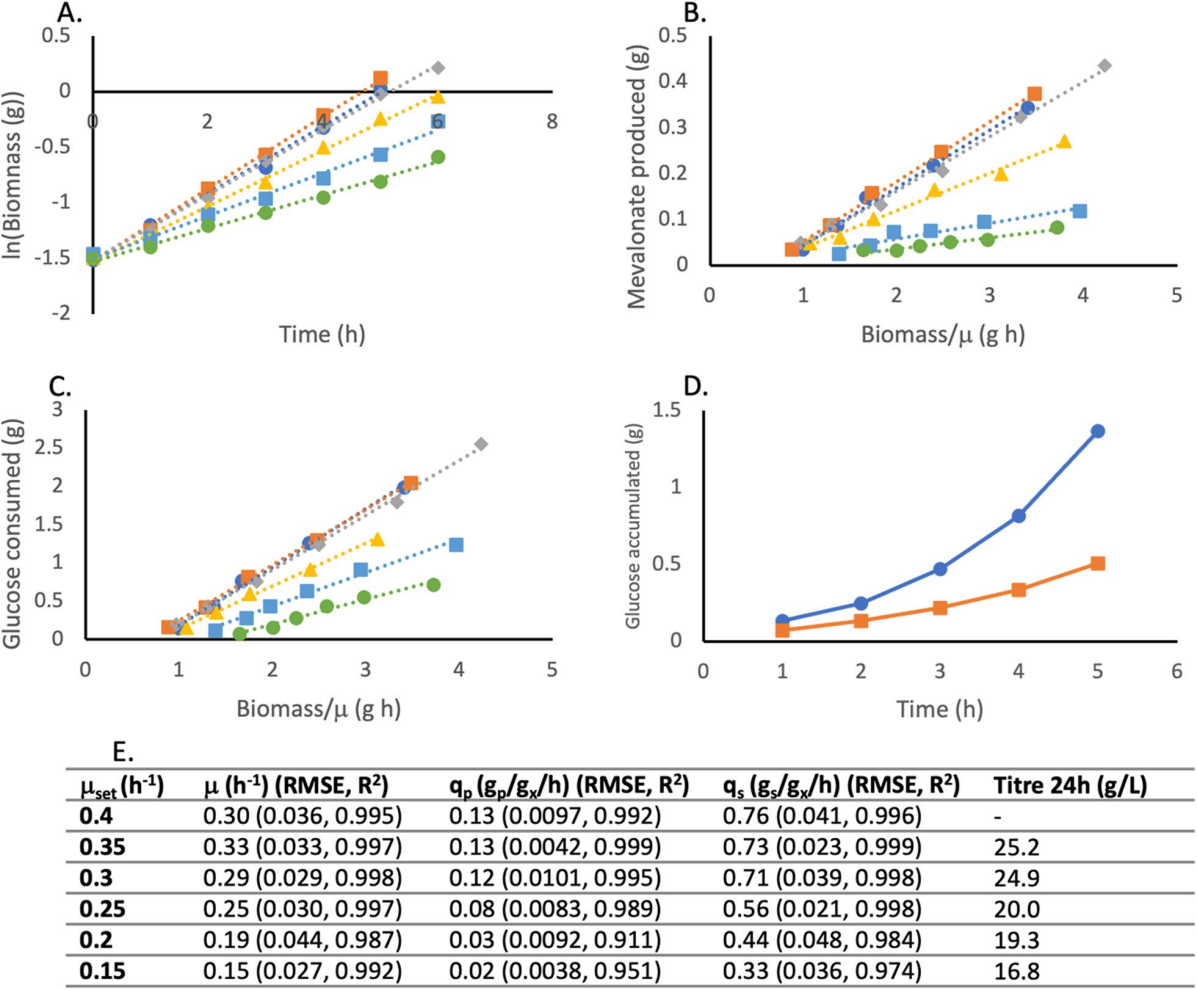
Mevalonate production by KO_MvaES, during six parallel fed-batch fermentations with defined $\mu$_set_ values. Values normalised to t = 0, for the beginning of steady-state conditions after batch phase, once feed has begun. **A** Specific growth rate of fed-batch reactions. **B** Specific product formation rate (q_p_) of fed-batch reactions. **C** Specific substrate consumption rate (q_s_) of fed-batch reactions. **D** Amount of glucose present in the culture medium. **E** Table containing the values, root mean squared error (RMSE) and correlation coefficient (R^2^) of $\mu$, q_p_ and q_s_ for each $\mu$_set_, obtained from panels **A**-**C**, and titre after 24 h of feeding. Blue circles: $\mu$_set_ = 0.4 h^−1^, orange squares: $\mu$_set_ = 0.35 h^−1^, grey diamonds: $\mu$_set_ = 0.3 h^−1^, yellow triangles: $\mu$_set_ = 0.25 h^−1^, light blue squares: $\mu$_set_ = 0.2 h^−1^, green circles: $\mu$_set_ = 0.15 h^−1^

Using feeding rates calculated from Fig. 6, all six fed-batch fermentations achieved constant specific growth rates (Fig. 7 A). Similarly to BW_MvaES, the highest two $\mu$_set_ values led to slightly lower than expected $\mu$, due to the accumulation of glucose in the culture medium. However, unlike BW_MvaES, acetate was not detectable during any of the fermentations. Again, the highest $\mu$_set_ led to the highest q_s_ and q_p_, and vice versa (Fig. 7 B and C). The highest q_p_ value was 0.13 g_p_/g_x_/h at a $\mu$_set_ of 0.4 or 0.35 h^−1^.

### Acetate-free growth coupling of mevalonate production

**Fig. 8:**
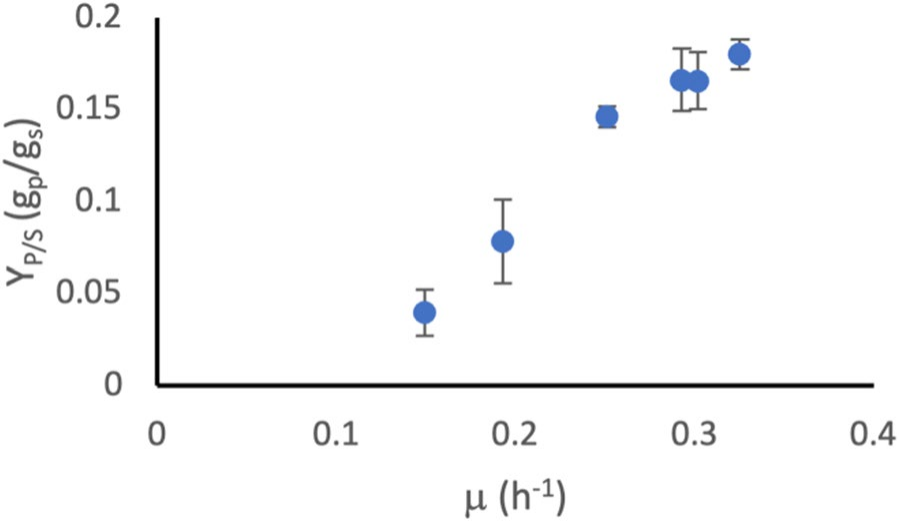
The relationship between Y_P/S_ and $\mu$ for KO_MvaES. Error estimated by error propagation of q_p_ and q_s_ RMSE values

The highest Y_P/S_ for KO_MvaES was 0.18 g_p_/g_s_, achieved at a $\mu$ of 0.34 h^−1^ (Fig. 8). At growth rates lower than this, Y_P/S_ decreases, with an almost a fivefold lower q_s_ to q_p_ ratio (0.039 g_p_/g_s_), at a $\mu$ of 0.15 h^−1^.

## Discussion

Reporting product titres in g/L is standard practice in academia [10, 11, 21, 22]. However, these values provide little relevant context when considering scaling up a process, where time and amount of substrate are both important factors in considering scaling costs. This is because whilst Cap-EX of a pilot or industrial plant are important, the ongoing Op-Ex, such as substrate provision, are far more important as they are paid for over the lifetime of the plant [23]. The only way to recover these costs is by production of the compound of interest, therefore the time required, and amount of product made are vital. Here, we studied the production of mevalonate in terms of rates, in the form q_p_ and q_s_. The units, g/g_x_/h, are related to product/substrate, biomass, and time. These may inform a more detailed TEA, based on the timescale of a production run and feedstock requirements rather than just endpoint titre.

Products made during fermentation, directly from metabolic substrates, are often considered to be ‘growth coupled’ [24]. Growth coupling results from cells having a ‘choice’ over how to utilise each unit of substrate. The substrate may be used by the cell to make a compound of interest, generate biomass or contribute towards cellular maintenance (ms, Eq. 1), such as pumping ions to maintain intracellular pH, producing side products or regeneration of proteins. For calculating F_t_ (Eq. 1), a literature value of 0.06 g_s_/g_x_/h was used. However, by plotting q_s_ against $\mu$ for BW25113_MvaES (Additional File 1: Fig. 1), the observed ms of 0.053 g_s_/g_x_/h can be obtained from the intercept. From this plot it is also possible to determine the Y_x/s_, through the inverse of the gradient (0.47 g_x_/g_s_, RMSE = 0.009). This value is significantly lower than the estimate of 0.5 g_x_/g_s_ (Fig. 2 A) (Student’s t-test p = 0.0102). The Y_x/s_ of KO_MvaES is significantly lower than that of BW25113_MvaES (Additional file 1: Fig. 2) (0.38 g_x_/g_s_, RMSE = 0.012) (Student’s t-test p < 0.05), indicating the knockouts are affecting the conversion of substrate to biomass.

Equation 1 was successful in generating steady-state growth conditions, as shown by the high R^2^ values of the linear growth rate plots (Figs. 3 A and 7 A). Equally, the linearity and growth rate dependence of q_p_, confirms that mevalonate production was growth coupled. As steady-state conditions are achieved, RMSE can give an estimate of experimental error, without the need for multiple runs at each growth rate. As the goal is to rapidly estimate important parameters at a low level of technology readiness, reducing the number of experimental runs is important in increasing speed and reducing costs, to make the process widely applicable. However, repeating the fermentation of BW_MvaES at a $\mu$_set_ of 0.3 h^−1^ (Additional file 1: Fig. 3), shows no significant difference in the values of q_s_ (Student’s t-test p = 0.9), q_p_ (Student’s t-test p = 0.95) or Y_P/S_ (Student’s t-test p = 0.72), demonstrating the reproducibility of the process.

The growth-coupling relationship is usually growth rate dependent, where cellular resources and cell metabolism at different growth rates may affect the partitioning of resources and therefore the proportion of substrate diverted to product. The relationship is strain and condition specific, and changes through genetic engineering or modification of growth conditions can affect the outcome.

Here, we studied growth-coupling relationships in the form of Y_P/S_ at different growth rates (Figs. 4 and 8), for BW_MvaES and KO_MvaES under defined growth conditions. The growth-coupling relationship for both strains aligned with previous observations [25] that higher growth rates provide increased acetyl-CoA availability and therefore lead to increased mevalonate production, as shown by the significant decrease in Y_P/S_ between $\mu$ of 0.33 h^−1^ and $\mu$ bellow 0.3 h^−1^ (Student’s t-test p < 0.05).

Y_P/S_ indicates the amount of product formed per g substrate. Calculating this simple measure could already inform that a process is unlikely to be economically viable if substrate costs exceed the expected revenue from product sold. In the strains assessed here, both achieved the same maximum Y_P/S_ of 0.18 g_p_/g_s_, indicating that both strains had the same maximum efficiency. If these strains had been studied in a typical manner, looking only at titre, it may not have been apparent that the knockouts did not affect efficiency. To achieve maximum efficiency, both strains must be grown at a rate of approximately 0.34 h^−1^. Knowledge of this can inform process design to ensure substrate feeding maintains growth rates favourable for maximum yield. In addition, both strains had the same maximum productivity, with no significant difference in q_p_ value (Student’s t-test p > 0.05), indicating that the rate of product formation was also the same. Knockouts did not significantly affect q_s_ at either $\mu$_set_ 0.3 h^−1^ or 0.15 h^−1^ (Student’s t-test p > 0.05).

The landscape of the growth-coupling relationship gives an indication of the robustness of the strain to changes in growth rate. This is an important consideration in scale-up, where mass transfer and mixing effects may lead to a heterogeneous population within the reactor, with not all cells growing under optimal conditions for product formation [26]. Comparing BW_MvaES and KO_MvaES, as $\mu$ decreases from the optimum the KO_MvaES was more adversely affected with lower Y_P/S_ values (0.04 g_p_/g_s_ versus 0.09 g_p_/g_s_ at a $\mu$ of 0.15 h^−1^). The low Y_P/S_ values at low growth rates represent a 3- to 5-fold higher minimum selling price for product, based on substrate cost per product, when compared to the same strain achieving its maximum efficiency. This would not have been identified by studying the endpoint titre for BW_MvaES and KO_MvaES.

Whilst both strains achieved the same maximum productivity, KO_MvaES achieved this without the formation of acetate as a byproduct. Acetate formation is undesirable as it can inhibit growth from around 5 g/L, which could prevent the desired growth rate being achieved at higher cell densities [27]. Additionally, the OP-Ex of DSP are one of the most significant factors affecting the commercialisation of fermentation based routes to organic acids because separating dilute mixed products is challenging [17]. Removal of acetate as a significant byproduct can help reduce the costs associated with DSP [16]. In industry, one common approach to reducing acetate formation is to reduce growth rate [28]. However, by reducing growth rate the productivity of BW_MvaES would be reduced, as can be seen by the lower Y_P/S_ and q_p_ values (Figs. 3, 4).

Typically, productivities are studied during steady-state growth in a chemostat [29]. However, the time, workload and practicality of studying multiple growth rates, makes rapidly determining a growth-coupling relationship challenging. More recently, accelerostat cultivation has been used, where increasing dilution rates are used to generate quasi-steady state conditions, until washout occurs [30]. However, this requires the use of specialist reactors with the ability to maintain a constant culture volume. Additionally, these systems can only maintain steady-state conditions for a short period of time and are therefore very different to how an industrial process would be carried out.

Fed-batch is a common mode of industrial fermentation. Studying steady-state conditions during fed-batch, allowed parallel bioreactors (ambr250) to be utilised, and the growth-coupling relationship to be calculated in a single experimental run. By parallelising the fermentations, growth-coupling relationships were calculated more rapidly than would have been possible during a chemostat study or by carrying out multiple fed-batch runs in a single, larger-scale fermenter. Parallelisation allows characterisation to be accelerated in a standardised manner, which is of interest in both the academic setting, and the SME (small and medium-sized enterprise) world, where time is money, and currently much de-risking of scale-up is carried out. Whilst demonstrated for mevalonate production, this process could readily be applied to many bioprocesses irrespective of product or production strain, for which growth-coupling relationships would likely differ and could lead to more efficient and commercially viable processes.

As studies here assumed a constant volume (Eq. 1), the rates are only true if the volume of feed added is significantly lower than the initial volume and therefore, only initial rates remain constant, and as feed volume increases, $\mu$ tends to decrease.

The purpose of this study was not to achieve maximum titres, but instead strain characterisation which can aid understanding and future process development. Since titre is intrinsically linked to production time, maximum titres would be achieved by maintaining a growth rate which gives the highest product yield coefficient, until high cell densities prevent further growth. This could be achieved by using a modified feeding equation, which adds a dilution factor to account for changes in volume from feed addition (Additional file 1: Eq. 1). However, the maximum oxygen transfer rate of our system is too low to support the high oxygen uptake rates required by the high growth rates and high cell densities needed to demonstrate this further.

Another metric commonly used to assess bioprocesses is overall volumetric productivity (g_p_/L/h). This is a characteristic of the process. It considers the reaction time and maximum titre and is independent of scale. Under the current feed rates, mevalonate concentrations of over 25 g/L were reached in less than 24 h (1.04 g/L/h) (Figs. 3 and 7). However, these titres were reached after the initial steady-state conditions (5 h for $\mu$_set_ = 0.4). Therefore, for much of the fermentation, lower growth rates with less desirable Y_P/S_ were present, reducing endpoint titre. Despite this, observed volumetric productivities were comparable to other studies of mevalonate production from glucose, where 47 g/L was reached in 50 h (0.94 g/L/h) [11], without the co-accumulation of acetate. Additionally, these titres were achieved by process design, with limited costly and time-consuming strain engineering, in just two rounds of fermentation (preliminary calculation of Y_x/s_, $\mu$_max_ and x_0,_ then simultaneous quantification of q_s_ and q_p_ across a range of $\mu$_set_), showing the importance of process design and demonstrating the ability of the process to rapidly achieve competitive titres.

## Conclusions

Here we report the use of parallel bioreactors to rapidly determine growth-coupling relationships of mevalonate production. By studying these in terms of productivity, we accessed industrially relevant insights, which would not be possible using traditional, titre-based reporting. This led to the development of a process capable of producing mevalonate at high rates without undesirable acetate formation and limited strain engineering. The process we developed could be applied to many fermentation products, to help to rapidly improve the efficiency of fermentation and to inform further process design and commercial feasibility studies.

## Materials and methods

### Construction of mevalonate-producing strain

*mvaE* and *mvaS* from *Enterococcus faecalis* were cloned into pBbS1k [31]. The resultant plasmid, pBbs1k_MvaES (Additional file 1: Fig. 4), was used for mevalonate production. This was transformed into electrocompetent *E. coli* BW25113, giving strain BW_MvaES.

### Parallel bioreactor fermentation

Fermentations were carried out in a Sartorius AMBR250 bioreactor system. Fermentations contained 150 mL of batch medium. Batch medium contained M9 salts (Sigma Aldrich), 4 g/L glucose, 2 mM MgSO_4_, 0.1 mM CaCl_2_, 50 $\mu$M kanamycin and trace elements (100 × 5 g/L EDTA.2Na.2H_2_O, 830 mg/L FeCl_3_.6H_2_O, 84 mg/L ZnCl_2_, 13 mg/L CuCl_2_.2H_2_O, 10 mg/L CoCl_2_.6H_2_O, 10 mg/L H_3_BO_3_, 1.6 mg/L MnCl_2_.4H_2_O). The temperature was maintained at 32 °C and the pH controlled at 7 via automatic addition of 15% NH_4_OH. Dissolved oxygen (DO) concentration was maintained at 30% by a stirring cascade, with a constant airflow of 1 v/v/min. Expression of *mvaES* was induced at approximately OD_600_ 1 by the addition of 250 $\mu$M IPTG. Feeding began after DO spiked above 50%, indicating starvation had occurred. Feed solution contained 500 g/L glucose, M9 salts, 5 × trace elements, 5 mM MgSO_4_, 250 $\mu$M IPTG, 50 $\mu$M kanamycin.

### Sample analysis

Quantification of glucose, mevalonate and acetate was carried out by HPLC. A REZEX ROA column was used, with an aqueous 5 mM H_2_SO_4_ mobile phase. The chromatography was run isocratically at 0.6 mL/min, at 55 °C for 30 min with RI detection. Analytical standards were used to generate curves for quantification (Additional file 1: Fig. 5).

### Knocking out acetate production

Acetate producing genes were knocked out of *E. coli* BW25113 using CRISPR–Cas12a, as used by Jervis et al*.* (20). 500 bp homology arms upstream and downstream of *poxB* and the *ackA_pta* operon were synthesised as gblocks by IDT and used to construct pTarget_AckApta (Additional file 1: Fig. 6) and pTarget_poxB (Additional file 1: Fig. 7). The knockout strain was made electrocompetent and transformed with pBbS1k_MvaES, to give the production strain KO_MvaES.

### Strain screening

Strains were screened in 250-mL flasks, containing 50 mL LB ± 20 g/L glucose. *mvaES* expression was induced at OD_600_ 1 with 250 $\mu$M IPTG. Cultures were grown at 32 °C for 16 h in a 200 rpm shaking incubator.

## Supplementary Information

Additional file 1. 

## Data Availability

The genome sequence of *E. coli* BW25113 is available on GenBank (GCA _ GCA_016811895).

## Abbreviations

TEA: Techno economic analysis
CAP-EX: Capital expenditure
OP-EX: Operating expenditure
q_p_: Specific product formation rate
μ: Specific growth rate
q_s_: Specific substrate consumption rate
Y_p/s_: Product yield coefficient
AcCoA: Acetyl-CoA
HMG-CoA: 3-Hydroxy-3-methylglutaryl-CoA
MVA: Mevalonate
AACT: Acetoacetyl-CoA thiolase
HMGR: 3-Hydroxy-3-methylglutarate reductase
HMGS: 3-Hydroxy-3-methylglutarate synthase
Pta: Phosphate acetyltransferase
AckA: Acetate kinase
PoxB: Pyruvate oxidase
TCA: Tricarboxylic acid
DSP: Downstream processing
KO: Knockout
