# Supplementary material for: A parallel bioreactor strategy to rapidly determine growth-coupling relationships for bioproduction: a mevalonate case study
Source: Biotechnol Biofuels Bioprod. 2025 Jan 17;18:6. doi: 10.1186/s13068-024-02599-x (PMC11742524; doi:10.1186/s13068-024-02599-x)
Supplement: Supplementary file 1 — Additional file 1. [file 13068_2024_2599_MOESM1_ESM.docx]

# Additional File 1

# A parallel bioreactor strategy to rapidly determine growth coupling relationships for bioproduction: a mevalonate case study

Alec Banner^1^, Joseph Webb^1,†^ & Nigel Scrutton^1*^

^1^Manchester Institute of Biotechnology, The University of Manchester, 131 Princess Street, Manchester, M1 7DN, United Kingdom

^†^Current Address Imperagen Ltd, Manchester Science Park, Manchester, M15 6SE, United Kingdom

*Corresponding author

$$F_{tv}=\left( \frac{V_{0}+\left( ms+\frac{\mu_{set}}{Y_{\frac{x}{s}}} \right).\left( \frac{X_{0}.V_{0}}{w_{in}} \right).e^{\mu_{set}t}}{V_{0}+\left( ms+\frac{\mu_{set}}{Y_{\frac{x}{s}}} \right).\left( \frac{X_{0}.V_{0}}{w_{in}} \right)} \right).\left( \left( ms+\frac{\mu_{set}}{Y_{x/s}} \right).\left( \frac{X_{0}.V_{0}}{w_{in}} \right).e^{\mu_{set}t} \right)$$

**Equation 1 Modified feeding strategy, accounting for dilution, to maintain μ_set_**. F_tv_ = time and volume dependent feed rate, ms = maintenance value, x_0_ = initial biomass concentration (g/L), v_0_ = initial culture volume (l), ω_in_ = concentration of rate limiting substrate in feed, t = time (h)


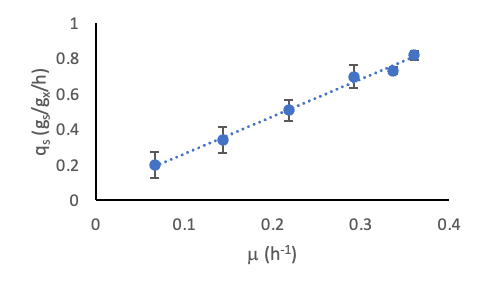


**Figure 1 Plot of q_s_ against growth rate used for the quantification of the ms of BW25113_mvaES.** Gradient = 2.103, Intercept = 0.053, R^2^ = 0.992, Error bars indicate q_s_ RMSE.


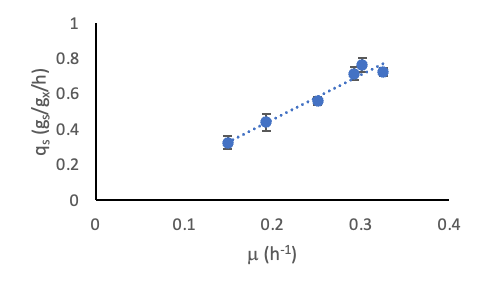


**Figure 2 Plot of q_s_ against growth rate used for the quantification of the ms of KO_mvaES.** Gradient = 2.528, Intercept = -0.05, R^2^ = 0.963, Error bars indicate q_s_ RMSE.


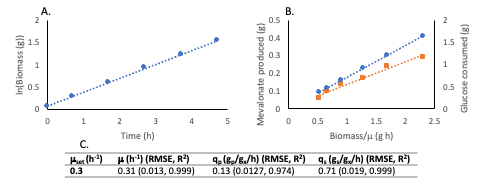


**Figure 3** **Mevalonate production by BW_MvaES, during fed-batch fermentations with defined μ_set_ value of 0.3 h^-1^**. Values normalised to t=0, for the beginning of steady-state conditions after batch phase, once feed has begun. A) Specific growth rate of fed-batch reactions. B) Specific product formation rate (q_p_) (orange squares) and Specific substrate consumption rate (q_s_) (blue circles) of fed-batch reactions. C) Table containing the values, RMSE and R^2^ of μ, q_p_ and q_s_, obtained from panels A & B. Y_P/S_ = 0.18 g_p_/g_s_.

A)


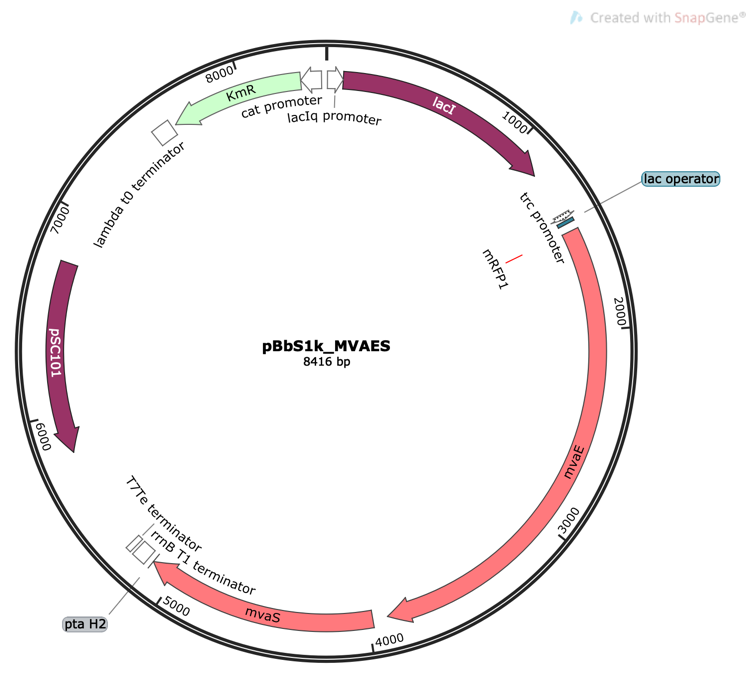


B)

atgaaaaccgtggtgattattgatgcactgcgtaccccgattggtaaatacaaaggtagcctgagccaggttagcgcagttgatctgggcacccatgttaccacccagctgctgaaacgtcatagcaccattagcgaagaaattgatcaggtgatttttggcaatgttctgcaggcaggtaatggtcagaatccggcacgtcagattgcaattaatagcggtctgagccatgaaattccggcaatgaccgttaatgaagtttgtggtagcggtatgaaagcagttattctggcaaaacagctgatccagctgggcgaagccgaagttctgattgccggtggtattgaaaatatgagccaggcaccgaaactgcagcgtttcaattatgaaaccgaaagctatgatgcaccgtttagcagcatgatgtatgatggtctgaccgatgcatttagcggtcaggcaatgggtctgacagcagaaaatgttgcagaaaaatatcatgtgacccgtgaagaacaggatcagtttagcgttcatagccagctgaaagcagcacaggcacaggccgaaggtattttcgcagatgaaattgcaccgctggaagttagcggcaccctggttgaaaaagatgaaggtattcgtccgaatagcagcgttgaaaaactgggtacactgaaaacggtgtttaaagaagatggcaccgttaccgcaggcaatgcaagtaccattaatgatggtgcaagcgcactgattattgccagccaagaatatgccgaagcacatggtctgccgtatctggcaattattcgtgatagcgttgaagttggtattgatccggcatatatgggtattagcccgattaaagcaattcagaaactgctggcacgtaatcagctgaccaccgaagaaatcgacctgtacgaaattaatgaagcatttgccgcaaccagcattgttgttcagcgtgaactggcactgccggaagaaaaagttaacatttatggcggtggcatcagcctgggtcatgcaattggtgcaaccggtgcacgtctgctgaccagcctgagctatcagctgaatcagaaagagaaaaaatacggcgttgcaagcctgtgtattggtggtggcctgggtctggcaatgctgctggaacgccctcaacagaaaaaaaacagccgtttttatcagatgagtccggaagaacgtctggccagcctgctgaatgaaggtcagattagcgcagataccaaaaaagaatttgaaaacaccgcactgagcagccagattgccaaccacatgattgaaaatcagatcagcgaaaccgaagtgccgatgggtgttggtctgcatctgaccgtggatgaaacggattatctggttccgatggcaaccgaagaaccgagcgttattgcagccctgagcaatggtgcaaaaattgcacagggctttaaaaccgtgaatcagcagcgtctgatgcgtggtcagattgttttttatgatgttgccgatgcagaaagcctgattgatgaactgcaggttcgtgaaacagaaattttccagcaggcagaactgagttatccgagcattgttaaacgcggtggtggtctgcgtgatctgcagtatcgtgcatttgatgaaagttttgttagcgtggattttctggtggatgttaaagacgcaatgggtgccaatattgttaatgcaatgctggaaggtgttgccgaactgtttcgtgaatggtttgcagaacaaaaaatcctgtttagcatcctgagtaactatgccaccgaaagcgttgttaccatgaaaacagcaattccggttagccgtctgagcaaaggtagtaatggtcgtgaaattgccgaaaaaattgttctggcaagccgttatgccagcctggatccgtatcgtgccgttacccataataaaggtattatgaatggcattgaagcagttgtgctggccaccggtaatgatacccgtgcagttagcgcaagctgtcatgcatttgcagttaaagaaggtcgttatcagggtctgaccagctggaccctggatggtgagcagctgattggtgaaattagcgttccgctggcactggcaaccgttggtggtgccaccaaagttctgccgaaaagccaggcagcagccgatctgctggcagttaccgatgcaaaagaactgagccgtgttgttgcagcagttggtctggcacagaatctggcagcactgcgtgcactggttagcgaaggcattcagaaaggtcacatggcactgcaggcacgttcactggccatgaccgtgggtgcgaccggtaaagaagttgaagccgttgcacagcaactgaaacgccagaaaacaatgaatcaggatcgtgccctggcaattctgaatgatctgcgtaaacagtaa

C)

atgaccattggcatcgacaaaatcagcttttttgttccgccttactatatcgacatgaccgcactggccgaagcacgtaatgttgatccgggtaaatttcatattggtattggtcaggatcagatggccgttaatccgattagccaggatattgttacctttgcagcaaatgcagcagaagcaattctgaccaaagaagataaagaagccatcgatatggttattgttggcaccgaaagcagcattgatgaaagcaaagcagccgcagttgttctgcatcgtctgatgggtattcagccgtttgcacgtagctttgaaattaaagaaggttgttacggcgcaaccgcaggtctgcagctggcaaaaaatcatgttgcactgcatccggataaaaaagttctggttgttgcagcagatatcgccaaatatggtctgaatagcggtggtgaaccgacccagggtgccggtgcagttgcaatgctggttgcaagcgaaccgcgtattctggcactgaaagaggataatgttatgctgacgcaggatatctatgatttttggcgtccgaccggtcatccgtatccgatggttgatggtccgctgagcaatgaaacctatattcagagctttgcacaggtgtgggatgaacataaaaaacgtaccggtctggatttcgcagattatgatgcactggcctttcatattccgtataccaaaatgggtaaaaaagcactgctggcgaaaattagcgatcagaccgaagccgaacaagaacgtatcctggcacgttatgaagaaagcattatctatagccgtcgtgtgggtaatctgtataccggtagcctgtatctgggtctgattagcctgctggaaaatgcaaccaccctgaccgctggtaatcagattggtctgtttagctatggtagcggtgccgttgcagaattctttaccggtgaactggttgcaggttatcagaatcatctgcagaaagaaacccatctggccctgctggataatcgtaccgaactgagcattgcagaatatgaagcaatgtttgcagaaaccctggataccgatattgatcagaccctggaagacgaattaaaatatagcattagcgccattaataacaccgtgcgtagctatcgtaattaa

**Figure 4 pPBs1k_MvaES.** A) Plasmid map of pPBs1k_MvaES. Generated by SnapGene Software (snapgene.com), B) MvaE nucleotide sequence, C) MvaS nucleotide sequence.


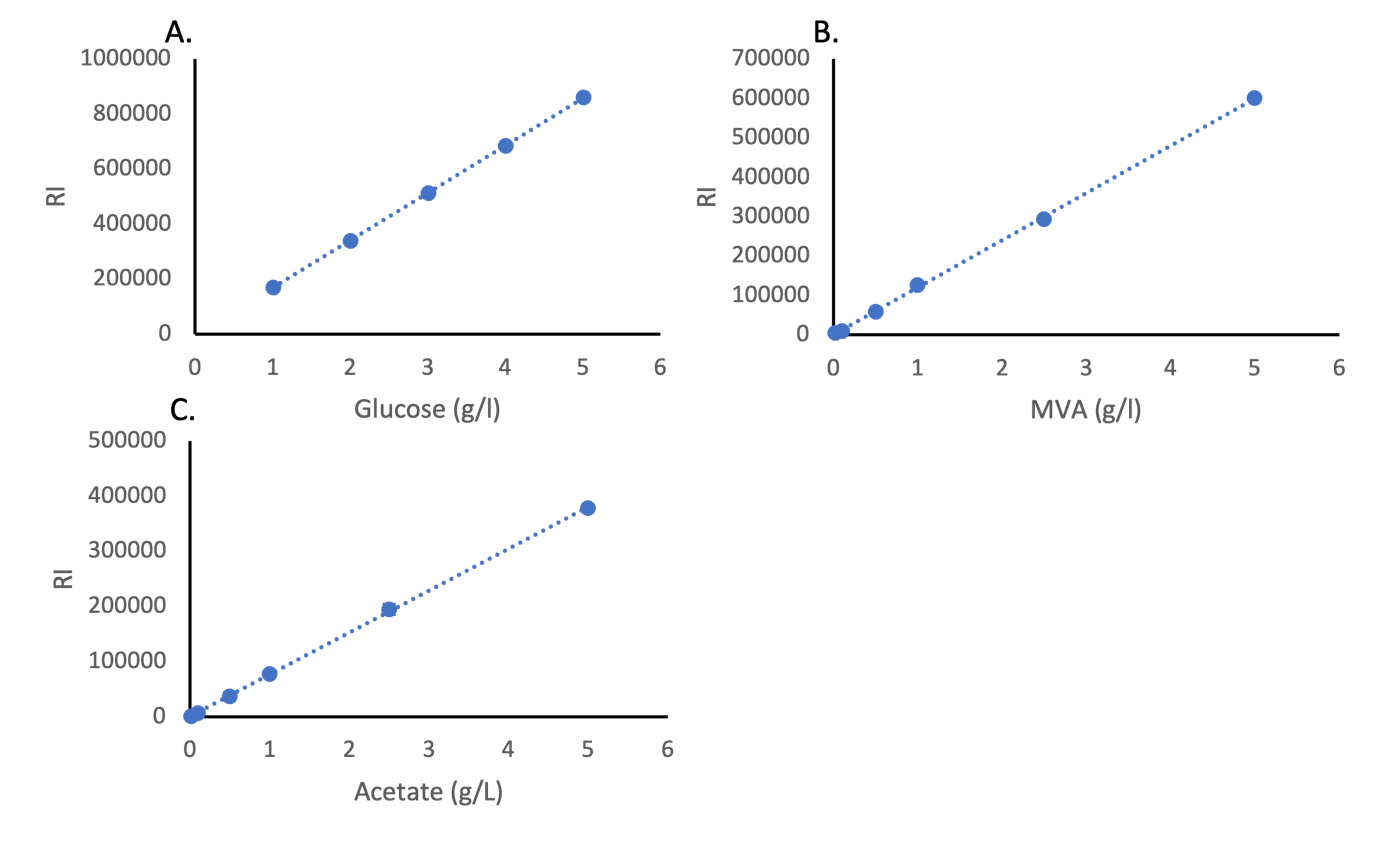


**Figure 5 Example standard curve used for HPLC quantification**. A) Glucose (y = 172521x – 4440), B) Mevalonate (y = 119661x + 686), C) Acetate (y = 76059x + 165).

A)


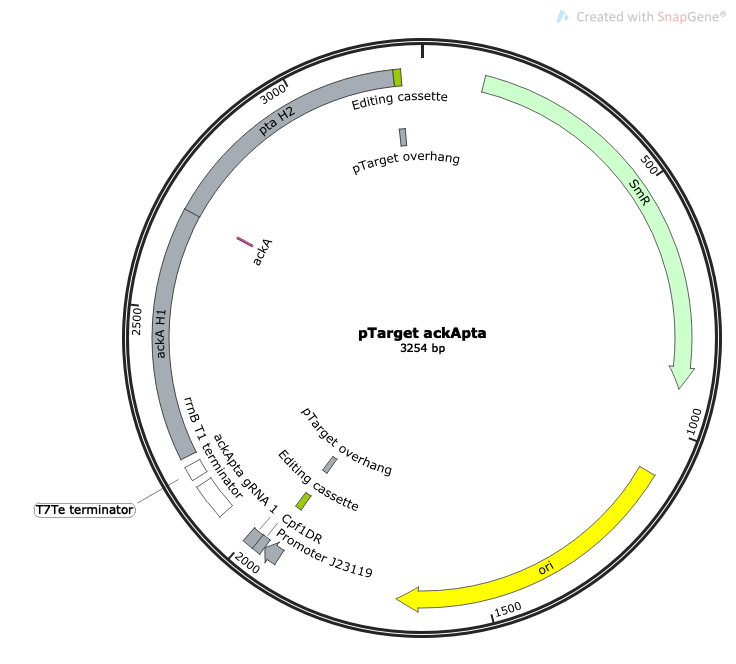


B)

cccagtctctgaactacgaaatc

C)

tcggcggcataaaacggatcgcataacgcgtcatcttgataacgcgattttcgacaaagaccggggcaaggcgtttttccagcggccacgtctttgagtaatgctgtccccggcgaaacaagctaaaaaaattaacagaacgattatccggcgttgacatgcttcacctcaacttcacatataaagattcaaaaatttgtgcaaattcacaactcagcgggacaacgttcaaaacattttgtcttccatacccactatcaggtatcctttagcagcctgaaggcctaagtagtacatattcattgagtcgtcaaattcatatacattatgccattggctgaaaattacgcaaaatggcatagactcaagatatttcttccatcatgcaaaaaaaatttgcagtgcatgatgttaatcataaatgtcggtgtcatcatgcgctacgctctatggctccctgacgtttttttagccacgtatcaattataggtacttccatg

D)

taatctcgtcatcatccgcagctttgcgctgcggatatctgaaccggaaataatcactatttccggttttttattctcttaatttgcattaatcctttctgattatcttgcttaactgcgctgcatcaatgaattgcgccatcccactttgcatacttaccactttgttttgtgcaagggaatatttgcgctatgtccgcaatcactgaatccaaaccaacaagaagatgggcaatgcccgatacgttggtgattatcttttttgttgctattttaaccagccttgccacctgggtagttccggtggggatgtttgacagtcaggaagtgcagtatcaggttgatggtcaaacaaaaacacgcaaagtcgtagatccacactcatttcgcattctgactaacgaagcaggcgaacctgagtatcaccgcgtacagctgttcacgacgggcgatgaacgcccgggcctgatgaacttcccgtttgaaggattaacctcagg

**Figure 6 pTarget for AckAPta operon knockout.** A) Plasmid map of pTarget_AckApta. Generated by SnapGene Software (snapgene.com), B) AckAPta gRNA. C) AckA H1 - AckA upstream Homology arm. D) Pta H2 - Pta downstream homology arm

A)


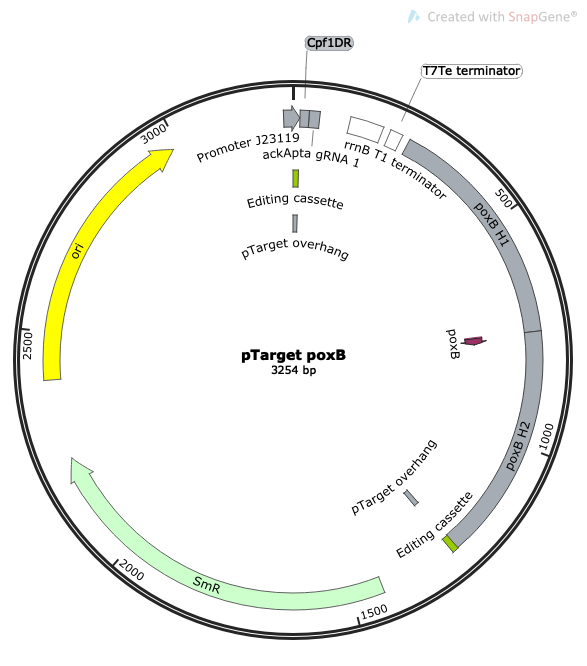


B)

ccgcgcagggcatgaacaatagg

C)

Gcccggctccgtatatggattgggtagagcaggaagtgaaagcgctcggcgtgacgcgtttctttaaagagaaattcttcaccccagtagcagaagcggcgaccagcggtctgaaattcaccaaactgcaaccggcacgagaattttacgccccggttggcaccacgctactggaggcgctggaaagcaataacgttccggttgtcgctgcctgccgtgcgggtgtttgcggctgctgtaagacaaaagtggtttccggtgaatatacggtgagcagcacaatgacgctgaccgacgccgaaatcgctgaaggttacgtactggcctgctcctgccatccgcagggggatttggttctcgcataatcgccttatgcccgatgatattcctttcatcgggctatttaaccgttagtgcctcctttctctcccatcccttccccctccgtcagatgaactaaacttgttaccgttatcacattcaggagatggagaaccatg

D)

Acaaactggctaaggtaaaaagggtggcatttcccgtcataataaggacatgccatgattgatttacgcagtgataccgttacccgaccaagccgcgccatgctcgaagcgatgatggccgccccggttggggacgacgtttacggagacgaccctaccgttaatgctctgcaggactacgcagcagagctttccggtaaagaagccgccatttttctgcctaccggcactcaggccaacctggtcgctctgctcagtcactgcgaacgcggcgaagagtatattgtcggtcaggccgcgcataactatctgtttgaagccggtggcgcggcggtgctgggcagtattcaaccgcaacccatagacgcggctgccgacggcacgctaccgctggataaagtggcgatgaaaatcaaacccgacgatatccatttcgcccgcaccaaattactcagtctggaaaacacccacaacggcaaagtgttgccgcgggaatacct

**Figure 7 pTarget for PoxB knockout.** A) Plasmid map of pTarget_poxB. Generated by SnapGene Software (snapgene.com), B) poxB gRNA. C) PoxB H1 - PoxB upstream Homology arm. D) PoxB H2 - PxoB downstream homology arm**.**
